# Effects of Isopropyl Alcohol, Tetrahydrofuran, Pyridine, and Acetonitrile on Surface Roughness, Surface Morphology, and Shear Bond Strength Between Composite Resin and Different Provisional Restorative Materials

**DOI:** 10.3390/dj14050309

**Published:** 2026-05-18

**Authors:** Nutchapol Thongsawas, Awutsadaporn Katheng, Santiphab Kengtanyakich, Hathairat Lekatana, Wisarut Prawatvatchara

**Affiliations:** 1 Department of Restorative Dentistry, Faculty of Dentistry, Naresuan University, Phitsanulok 65000, Thailand; 2 Department of Prosthodontics, Faculty of Dentistry, Chulalongkorn University, Bangkok 10330, Thailand

**Keywords:** provisional restoration, PMMA, 3D printing, solvents, isopropyl alcohol, tetrahydrofuran, pyridine, acetonitrile, shear bond strength

## Abstract

**Background/Objectives:** Provisional restorations are essential in prosthodontic treatment, and reliable intraoral repair is clinically important during extended interim use. This in vitro study evaluated the effects of organic solvent pretreatment on surface characteristics and shear bond strength (SBS) of CAD/CAM provisional restorative materials fabricated by milling, stereolithography (SLA), and digital light processing (DLP). **Methods:** Three materials were assigned to five surface treatment conditions: no solvent (control), isopropyl alcohol (IPA), tetrahydrofuran (THF), acetonitrile (ACN), and pyridine (PYR). After pretreatment, separate specimens were used for surface analysis and SBS testing. Surface roughness was measured by atomic force microscopy using arithmetic mean height (S_a_) and root mean square height (S_q_), and surface morphology was examined by scanning electron microscopy (SEM). For SBS testing, specimens were repaired using a universal adhesive and a flowable resin composite, followed by failure mode analysis. Data were analyzed using two-way ANOVA and Tukey’s post hoc test (α = 0.05). **Results:** Material type, solvent treatment, and their interaction significantly affected SBS, S_a_, and S_q_. The DLP material showed the highest SBS overall, with no significant differences among treatments. In the SLA material, ACN resulted in the lowest SBS, whereas PYR showed the highest mean value. In the milled material, THF, ACN, and PYR produced significantly higher SBS than the control and IPA groups. **Conclusions:** Within the limitations of this study, the effect of organic solvent pretreatment on repair performance was substrate-dependent.

## 1. Introduction

Provisional restorations are essential in prosthodontic treatment, providing protection, function, and esthetics during the interim phase prior to definitive rehabilitation. With increasingly complex treatment plans and the widespread adoption of digital workflows, provisionals are often required to function for extended periods, which increases the incidence of mechanical and adhesive failures such as fracture, chipping, and debonding [1,2]. These complications highlight the clinical importance of reliable intraoral repair strategies.

Traditionally, provisional restorations have been fabricated from polymethyl methacrylate (PMMA) using conventional or subtractive milling techniques. More recently, additive manufacturing technologies, including stereolithography (SLA) and digital light processing (DLP), have been increasingly adopted because of their efficiency, accuracy, and material-saving advantages [3,4,5]. However, differences in polymer structure, filler content, degree of conversion, and surface characteristics among CAD/CAM provisional materials may influence their response to surface treatment and repair procedures [6,7,8]. Consequently, repair protocols established for milled PMMA may not be directly applicable to additively manufactured provisional materials.

Repair of provisional restorations is commonly performed using resin composite materials; however, achieving durable adhesion to aged provisional substrates remains challenging because of their highly polymerized nature and limited surface reactivity [9]. Various surface modification strategies have been proposed to improve repair bond strength, including mechanical and physicochemical approaches [8,10,11]. Among these, chemical surface pretreatment using organic solvents has gained attention because of its simplicity and ability to induce polymer chain swelling and partial surface softening, thereby enhancing micromechanical interlocking [12,13]. Previous studies have reported that solvents such as tetrahydrofuran and pyridine can improve bonding performance in polymer-based dental materials [14,15,16]. Isopropyl alcohol has also been investigated as a clinical reference solvent, though with comparatively limited polymer interaction [15]. In addition, acetonitrile was considered as a polar aprotic solvent whose Hansen solubility parameter profile suggests intermediate compatibility with polymethyl methacrylate, as characterized by its dispersion, polar, and hydrogen-bonding components [17]. Nevertheless, evidence regarding solvent-based pretreatment remains fragmented and is often limited to single material systems or fabrication methods.

To date, no standardized repair protocol has been established that accounts for both organic solvent chemistry and CAD/CAM fabrication method. In particular, comparative data evaluating CAD/CAM provisional restorative materials fabricated by milling, SLA, and DLP under identical solvent treatment conditions are lacking. Moreover, the relationship between solvent-induced surface modification and subsequent repair bond strength has not been fully elucidated.

Therefore, the purpose of this in vitro study was to evaluate the effects of different organic solvent surface pretreatments on the surface characteristics and shear bond strength of CAD/CAM provisional restorative materials fabricated by milling, SLA, and DLP. The null hypotheses were that (1) organic solvent surface pretreatment would not affect the surface roughness, surface morphology, and shear bond strength of repaired provisional restorations and (2) the effect of solvent pretreatment would not differ among CAD/CAM provisional materials fabricated by different methods.

## 2. Materials and Methods

### 2.1. Study Design

**Figure 1:**
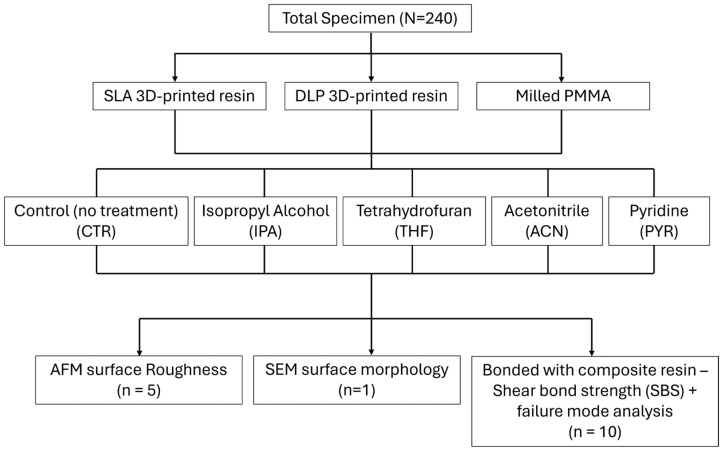
Experimental design and specimen allocation.

This in vitro study evaluated the effects of organic solvent surface treatment on CAD/CAM provisional restorative materials fabricated using different manufacturing techniques. Three CAD/CAM provisional restorative materials fabricated by milling (MIL), SLA, and DLP were investigated. Five surface treatment conditions were assessed: no treatment (control), isopropyl alcohol, tetrahydrofuran, acetonitrile, and pyridine. The outcomes included surface roughness, surface morphology, shear bond strength (SBS), and failure mode analysis, following established in vitro bonding test principles (Figure 1) [14,18,19,20].

### 2.2. Specimen Preparation

A total of 240 specimens (10 × 10 × 3 mm) were fabricated and allocated into three material groups [21]. Within each subgroup, separate specimens were prepared for each evaluation: 10 specimens for shear bond strength (SBS) testing and failure mode analysis, 5 specimens for surface roughness analysis, and 1 specimen for SEM surface morphology evaluation. Thus, different specimens were used for mechanical testing, roughness analysis, and SEM observation. For the additive manufacturing groups, specimens were designed as rectangular blocks using 3D Builder software (version 20.0.4.0; Microsoft Corporation, Redmond, WA, USA) and fabricated using an SLA printer (Form 3; Formlabs, Somerville, MA, USA) and a DLP printer (ASIGA Composer; ASIGA, Sydney, Australia). All printed specimens were oriented at 0° with a layer thickness of 50 µm. Post-printing washing and post-curing procedures were performed strictly according to the manufacturers’ instructions.

For the subtractive manufacturing group, specimens were milled from prepolymerized PMMA blocks using a dry dental milling machine (DWX-52D; Roland DG Corporation, Hamamatsu, Japan). The milled blocks were subsequently sectioned to standardized final dimensions of 10 × 10 × 3 mm to match those of the 3D-printed specimens. The materials used in this study are presented in Table 1.

All specimens were embedded in autopolymerizing acrylic resin using pre-cut polyvinyl chloride (PVC) molds (15 mm height, 20 mm outer diameter) to facilitate handling during surface treatment and testing. Surface standardization was performed sequentially using silicon carbide abrasive papers with grit sizes of 120, 240, 400, 800, and 1000 under continuous water irrigation. Each polishing step was performed unidirectionally to minimize surface irregularities. Following polishing, all specimens were ultrasonically cleaned in deionized water for 10 min (Sonorex Super 10P; BANDELIN electronic GmbH & Co. KG, Berlin, Germany) and air-dried with oil-free compressed air. This standardized polishing protocol was used to provide a consistent baseline surface preparation across all specimens and to reduce the influence of initial surface irregularities before solvent treatment.

### 2.3. Surface Treatment Protocol

Specimens were randomly assigned to five surface treatment groups. The control group received no surface treatment. The solvents used were isopropyl alcohol (propan-2-ol; 99.8%; RCI Labscan Limited, Bangkok, Thailand), tetrahydrofuran (99.5%; Loba Chemie Pvt. Ltd., Mumbai, India), acetonitrile (99.8%; Loba Chemie Pvt. Ltd., Mumbai, India), and pyridine (99.5%; Loba Chemie Pvt. Ltd., Mumbai, India). All solvents were used as received without dilution. For the solvent-treated groups, 10 µL of the assigned solvent (isopropyl alcohol, tetrahydrofuran, acetonitrile, or pyridine) was dispensed onto the specimen surface using a calibrated micropipette. The solvent was actively applied with a microbrush for 30 s to promote uniform surface interaction. This procedure was repeated once to standardize solvent exposure time. Following solvent application, all specimens were rinsed with deionized water for 10 s to remove residual solvent and air-dried using oil-free compressed air. This protocol was modified from a previous study to induce controlled chemical surface modification while minimizing excessive material dissolution [15].

### 2.4. Surface Roughness Analysis

To quantify solvent-induced changes in surface topography, five additional specimens from each subgroup were prepared for surface roughness analysis. Atomic force microscopy (AFM) was performed using a scanning probe microscope (Flex-Axiom; Nanosurf, Liestal, Switzerland) operated with Nanosurf C3000 software (version 3.8.8.12; Nanosurf, Liestal, Switzerland) in dynamic force mode. The scan area was set at 50 × 50 µm, with a resolution of 256 points per line and a scan time of 0.78 s per line. Each specimen was scanned at three predefined locations (top, middle, and bottom regions) to account for surface heterogeneity [22]. Surface roughness parameters were calculated from the acquired topographical data, and mean values were used for statistical analysis. Representative three-dimensional AFM topographic images were selected from specimens with roughness values closest to the subgroup mean in order to qualitatively illustrate the surface topography of each experimental condition.

### 2.5. Surface Morphology Evaluation

Surface morphological changes induced by solvent treatment were qualitatively evaluated using scanning electron microscopy (SEM) (LEO 1455VP; Angstrom Scientific, Cambridge, UK). Representative specimens from each solvent-treated group were sputter-coated with gold prior to examination (coating thickness: 12.6 nm; coating time: 100 s; current: 18 mA). The specimens were then observed at magnifications of 500× and 2000× under an accelerating voltage of 3 kV and a working distance of 14 mm. SEM analysis was performed to identify micro-porosities, surface irregularities, and other surface changes associated with polymer modification.

### 2.6. Shear Bond Strength Test

Following surface treatment, a universal adhesive (Scotchbond™ Universal Plus; 3M ESPE, St. Paul, MN, USA) was applied to the treated specimen surface using an active rubbing motion for 20 s and gently air-dried for 5 s according to the manufacturer’s instructions [23]. A medical-grade polyvinyl chloride (PVC) microextension tube (DEHP-free) with an inner diameter of 1.16 mm, an outer diameter of 2.1 mm, and a height of 1.0 mm was then positioned perpendicular to the specimen surface, after which the adhesive was light-cured for 10 s [21].

A flowable resin composite (Filtek™ Supreme Flowable Restorative; 3M ESPE) was injected into the tube to form a cylindrical composite build-up and light-cured for 20 s. The tube was then carefully removed. The bonded area, calculated from the internal diameter of the tube, was 1.0568 mm^2^.

**Figure 2:**
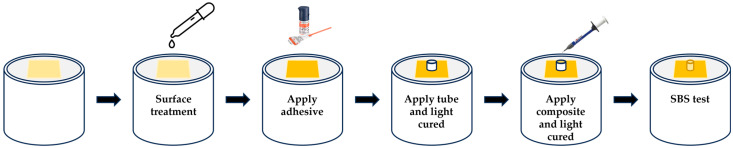
Schematic representation of the shear bond strength (SBS) test workflow. The specimen surface was treated, followed by application of a universal adhesive. A microextension tube was positioned and the adhesive was light-cured. Flowable resin composite was then placed into the tube and light-cured. After tube removal, the bonded specimen was subjected to SBS testing.

All bonded specimens were stored in distilled water at 37 °C for 24 h prior to mechanical testing [24]. Shear bond strength testing was performed using a universal testing machine (Instron ElectroPuls™ E1000; Instron, Norwood, MA, USA) equipped with a notched-edge loading blade. The blade was aligned parallel and as close as possible to the bonded interface, and load was applied at a crosshead speed of 0.5 mm/min until failure occurred. Shear bond strength (SBS), expressed in megapascals (MPa), was obtained by dividing the maximum load at failure (N) by the bonded area (mm^2^) [25,26]. The SBS specimen preparation and testing workflow is schematically illustrated in Figure 2.

### 2.7. Failure Mode Analysis

The debonded surfaces were examined under a stereomicroscope at 40× magnification to determine the mode of failure. Failures were classified descriptively as adhesive failure (failure entirely at the interface between the provisional material and resin composite), cohesive failure (failure entirely within the provisional material or resin composite), or mixed failure (combination of adhesive and cohesive failure) [27,28].

### 2.8. Statistical Analysis

Statistical analysis was performed using GraphPad Prism version 8.4.3 for Windows (GraphPad Software, San Diego, CA, USA). Compliance with parametric assumptions was assessed by examining the residuals of the fitted models. Homogeneity of variance across the material × solvent groups was additionally evaluated using the Brown–Forsythe test in IBM SPSS Statistics version 22 (IBM Corp., Armonk, NY, USA), and the detailed results are provided in Appendix A, Table A1. Because the untransformed shear bond strength (SBS) data violated the variance assumption, log-transformed values were used for two-way analysis of variance (ANOVA). For surface roughness, inferential analyses were additionally performed using log-transformed arithmetic mean height (S_a_) and root mean square height (S_q_) data to improve compliance with parametric assumptions. In each model, material type and solvent treatment were entered as fixed factors, and their interaction was also evaluated. Post hoc comparisons were performed using Tukey’s multiple comparisons test. Statistical significance was set at α = 0.05. SEM surface morphology and failure mode distributions were evaluated descriptively and were not subjected to inferential statistical analysis.

## 3. Results

Because the untransformed SBS data did not satisfy the homogeneity assumption, log-transformed values were used for two-way ANOVA. For surface roughness, inferential analyses were additionally performed using log-transformed arithmetic mean height (S_a_) and root mean square height (S_q_) data. Log transformation improved the suitability of the roughness data for parametric analysis; however, inspection of the residuals indicated some remaining deviation from normality, particularly for S_a_. Two-way ANOVA revealed significant effects of material, solvent treatment, and their interaction on log-transformed SBS, log-transformed S_a_, and log-transformed S_q_ (Table 2).

### 3.1. Surface Roughness

**Figure 3:**
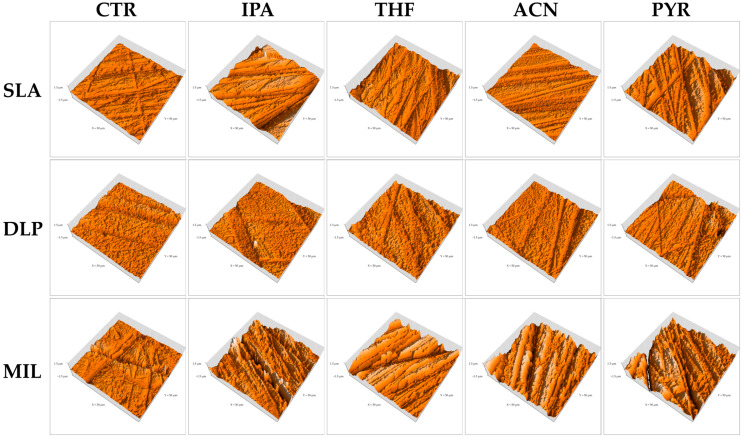
Representative three-dimensional AFM topographic images of the experimental groups. Rows represent material type (SLA, DLP, and MIL), and columns represent surface treatment (CTR, IPA, THF, ACN, and PYR). All panels were displayed with the same scan area of 50 × 50 µm and height range of −1.5 to 1.5 µm. The displayed images were selected from specimens with roughness values closest to the subgroup mean to qualitatively illustrate the surface topography associated with each experimental condition.

The surface roughness results are summarized in Table 3. For inferential analysis, log-transformed S_a_ and S_q_ data were used. Two-way ANOVA of the log-transformed data revealed significant effects of material type, solvent treatment, and their interaction for both roughness parameters, indicating that the effect of treatment depended on the material type. Representative three-dimensional AFM topographic images are shown in Figure 3. Although these images were evaluated qualitatively, they generally reflected the subgroup roughness patterns and provided visual support for the surface topography measured by AFM.

In the SLA group, the IPA subgroup showed significantly higher S_a_ than THF (*p* = 0.0045) and ACN (*p* = 0.0049), whereas no significant differences were found between IPA and CTR or PYR. A similar pattern was observed for S_q_, in which IPA was significantly higher than THF (*p* = 0.0098) and ACN (*p* = 0.0034), while the remaining pairwise comparisons were not significant. In the representative 3D AFM images, the SLA-IPA surface showed more pronounced irregular topography than the THF- and ACN-treated surfaces, consistent with the higher S_a_ and S_q_ values observed for IPA. The THF- and ACN-treated surfaces showed comparatively more regular ridge-like features, whereas the PYR-treated surface also showed pronounced surface features, consistent with its intermediate roughness values.

In the DLP group, no significant differences in S_a_ were observed among the treatment subgroups. For S_q_, only ACN and PYR differed significantly (*p* = 0.0209), whereas the remaining pairwise comparisons were not significant. The representative 3D AFM images of the DLP groups showed comparatively modest variation among treatments overall, consistent with the absence of significant differences in S_a_. Although ACN showed the lowest mean S_q_ and PYR showed the highest mean S_q_, the visual topographic differences among DLP subgroups remained limited.

In the MIL group, the control group showed significantly lower S_a_ than IPA (*p* = 0.0381), ACN (*p* = 0.0017), and PYR (*p* = 0.0051), whereas the difference between the control group and THF was not significant (*p* = 0.0592). For S_q_, the control group showed significantly lower values than ACN (*p* = 0.0034) and PYR (*p* = 0.0094), while no significant differences were found between the control group and IPA or THF. The representative 3D AFM images of the MIL groups showed more prominent ridge disruption and greater topographic irregularity after solvent treatment than in the control group, particularly in the IPA, ACN, and PYR groups. THF also showed a rougher surface appearance than the control, although the quantitative difference was not statistically significant.

### 3.2. SEM Observations

**Figure 4:**
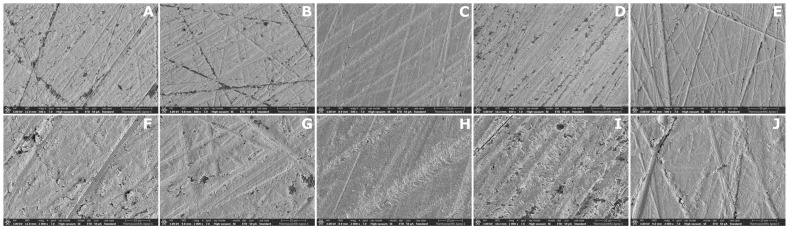
Representative SEM micrographs of the SLA groups at 500× (**upper row**) and 2000× (**lower row**) magnifications. (**A**,**F**) CTR; (**B**,**G**) IPA; (**C**,**H**) THF; (**D**,**I**) ACN; (**E**,**J**) PYR. Scale bars are shown in each micrograph.

**Figure 5:**
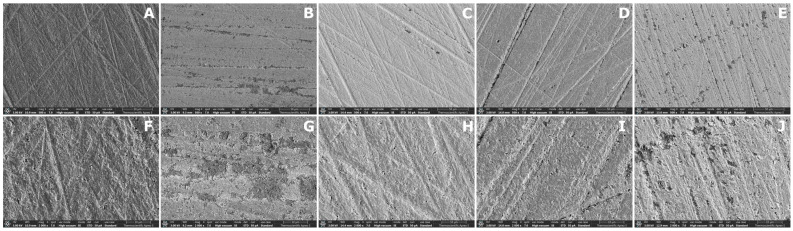
Representative SEM micrographs of the DLP groups at 500× (**upper row**) and 2000× (**lower row**) magnifications. (**A**,**F**) CTR; (**B**,**G**) IPA; (**C**,**H**) THF; (**D**,**I**) ACN; (**E**,**J**) PYR. Scale bars are shown in each micrograph.

**Figure 6:**
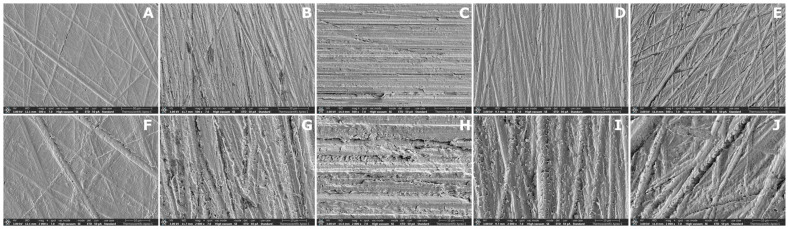
Representative SEM micrographs of the MIL groups at 500× (**upper row**) and 2000× (**lower row**) magnifications. (**A**,**F**) CTR; (**B**,**G**) IPA; (**C**,**H**) THF; (**D**,**I**) ACN; (**E**,**J**) PYR. Scale bars are shown in each micrograph.

Representative SEM images of each subgroup are shown in Figure 4, Figure 5 and Figure 6. Distinct surface morphologies were observed according to material type and solvent treatment.

SEM images of the SLA groups (Figure 4) showed preserved polishing grooves in all subgroups. The control specimens exhibited distinct linear grooves with scattered surface irregularities and porosities. Similar groove patterns were observed in the IPA- and ACN-treated groups, with no obvious alteration in groove continuity. In contrast, the THF- and PYR-treated specimens appeared smoother and more homogeneous, with fewer visible surface voids than the control, particularly in the THF group.

SEM images of the DLP groups (Figure 5) revealed generally well-preserved polishing grooves in the control, THF-, ACN-, and PYR-treated specimens. The control surface appeared relatively uniform, with limited surface irregularities and porosity. THF and ACN showed surface morphologies comparable to the control, whereas PYR exhibited slightly more visible surface voids. Among all DLP subgroups, the IPA-treated specimens showed the greatest surface alteration, with less distinct groove definition, increased porosity, and a less organized surface pattern.

SEM images of the MIL groups (Figure 6) showed that the control specimens had sharply defined polishing grooves and a relatively smooth surface. Following solvent treatment, the groove margins became less distinct and the surfaces appeared more irregular than the control. At higher magnification, all treated MIL subgroups showed disrupted groove continuity and increased heterogeneity along the groove edges. These surface changes were most evident in the THF-, ACN-, and PYR-treated groups.

### 3.3. Shear Bond Strength

**Figure 7:**
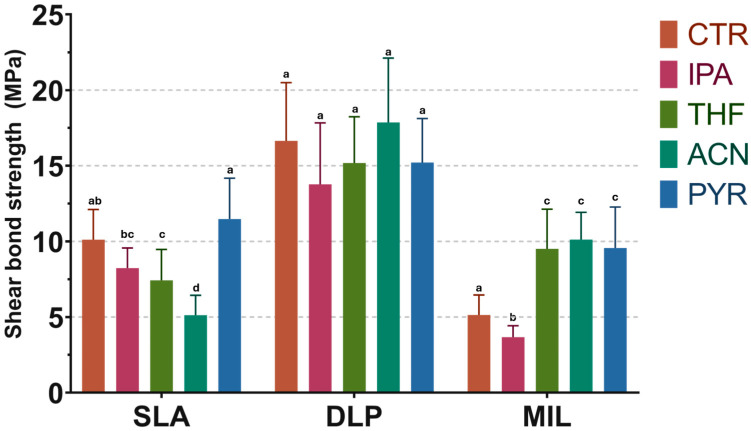
Shear bond strength (SBS) values of the experimental groups. Within each material, bars sharing the same lowercase letter are not significantly different according to Tukey’s multiple comparison test of the log-transformed data (*p* > 0.05). Error bars indicate standard deviations.

The shear bond strength (SBS) results are shown in Figure 7, and the complete mean ± SD values for all subgroups are presented in Appendix A, Table A2. In the SLA group, mean SBS values ranged from 5.13 MPa in ACN to 11.48 MPa in PYR. The ACN subgroup showed significantly lower SBS than all other SLA subgroups, whereas PYR showed significantly higher SBS than THF and IPA. The control group showed an intermediate mean SBS of 10.12 MPa and was significantly higher than THF (7.43 MPa), but not significantly different from IPA (8.24 MPa) or PYR.

In the DLP group, SBS values were the highest overall, ranging from 13.77 MPa in IPA to 17.86 MPa in ACN. However, no significant differences were found among the treatment subgroups.

In the MIL group, mean SBS values ranged from 3.67 MPa in IPA to 10.12 MPa in ACN. The control group (5.14 MPa) and IPA group showed the lowest SBS values, whereas THF (9.51 MPa), ACN (10.12 MPa), and PYR (9.57 MPa) showed significantly higher SBS. No significant differences were observed among THF, ACN, and PYR.

### 3.4. Failure Mode Evaluation

**Figure 8:**
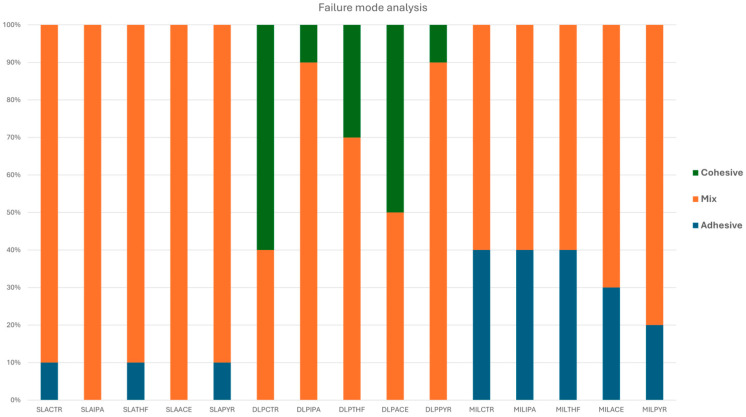
Distribution of failure modes in the experimental groups, expressed as percentages (*n* = 10 per group). Failure modes were classified as adhesive, cohesive, or mixed failure.

**Figure 9:**
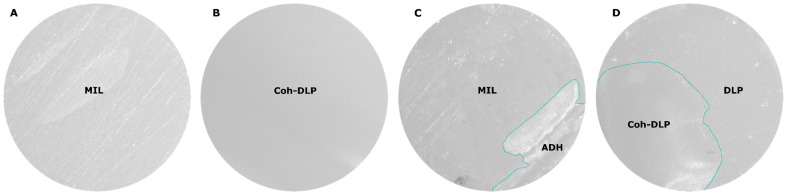
Representative stereomicroscopic images of failure modes after repair bond strength testing. (**A**) Adhesive failure on the milled PMMA substrate, showing exposure of the MIL surface. (**B**) Cohesive failure within the DLP material. (**C**) Mixed failure on the MIL substrate, showing exposed MIL surface and remaining adhesive. (**D**) Mixed failure on the DLP substrate, showing cohesive fracture within the DLP material and adhesive interfacial failure with exposed DLP surface. The cyan lines indicate the boundary between different failure regions. MIL, milled PMMA; DLP, digital light processing resin; ADH, adhesive remnant; Coh-DLP, cohesive fracture within the DLP material. Original magnification, 40×.

The distribution of failure modes is shown in Figure 8. Representative stereomicroscopic images illustrating adhesive, cohesive, and mixed failure modes are shown in Figure 9. In the SLA group, failures were predominantly mixed, with only a small proportion of adhesive failure in some subgroups and no cohesive failure observed.

In the DLP group, mixed failure was also the predominant failure mode. In addition, several DLP subgroups exhibited cohesive failure, particularly the control, THF, and ACN groups, whereas adhesive failure was not observed.

In the MIL group, both adhesive and mixed failures were observed. The control, IPA, and THF subgroups showed a greater proportion of adhesive failure, whereas the ACN and PYR subgroups showed a greater proportion of mixed failure. No cohesive failure was found in the MIL groups.

## 4. Discussion

The present study demonstrated that the effect of solvent pretreatment on repair bond strength was strongly material-dependent, as evidenced by the significant interaction between material type and solvent. Therefore, the repair effect of a given solvent could not be generalized across all CAD/CAM provisional restorative materials. Among the tested substrates, the DLP material showed the highest and most stable repair bond strength, whereas milled PMMA showed the clearest benefit from solvent pretreatment. In contrast, the SLA material exhibited a more selective response, indicating that repair performance was governed not only by solvent type but also by the chemical nature and microstructure of the substrate. Therefore, both null hypotheses were rejected.

A useful framework for interpreting these findings is polymer–solvent compatibility, commonly described by solubility-parameter theory. In general, solvents whose interaction characteristics are closer to those of the polymer are more likely to induce superficial swelling, softening, or partial dissolution, thereby affecting wetting, interfacial adaptation, and monomer diffusion at the repair interface [17,29]. PMMA solubility parameter is commonly reported around 22.7 MPa^1/2^, and THF appears relatively compatible with PMMA within the Hansen solubility framework (Appendix A, Table A3) [17]. By contrast, more polar solvents such as isopropyl alcohol and acetonitrile are expected to interact differently with PMMA-rich substrates. However, solubility-parameter matching alone cannot fully predict repair performance, because excessive solvent action may also weaken the superficial polymer layer and reduce effective stress transfer after repair [29,30].

This interpretation was most evident in the MIL group. ACN and PYR improved SBS relative to the untreated control, and these groups also showed significantly higher S_a_ and S_q_ values than the control. IPA significantly increased S_a_, whereas THF showed higher mean roughness values than the control but without a statistically significant difference for either S_a_ or S_q_. The representative 3D AFM images likewise showed more pronounced ridge disruption and topographic irregularity in the solvent-treated MIL groups than in the control, supporting the interpretation that solvent exposure altered the milled PMMA surface. SEM also showed progressive disruption of the original machined grooves after solvent application. Because milled PMMA is highly polymerized and relatively homogeneous, its untreated surface may provide limited chemical interaction with the repair resin, making it more dependent on solvent-mediated surface modification [31]. The favorable effect of THF and PYR is consistent with their stronger interaction with methacrylate-based matrices, while the effect of ACN suggests that, under the present conditions, it was still capable of producing beneficial superficial modification. Importantly, although isopropyl alcohol increased roughness, it did not produce the same bond-strength improvement as THF, acetonitrile, and pyridine, indicating that surface roughness alone was insufficient to explain repair performance.

The behavior of the DLP material differed from that of milled PMMA. The DLP groups showed the highest overall SBS, yet no significant differences were found among solvent pretreatments for SBS, and only limited differences were observed in surface roughness. No significant differences were found among treatments for S_a_, whereas for S_q_ only ACN and PYR differed significantly. The representative 3D AFM images also showed comparatively modest variation among DLP subgroups, consistent with the absence of significant differences in S_a_ and the limited pairwise difference observed for S_q_. Although ACN showed the lowest mean S_q_ and PYR showed the highest mean S_q_, these topographic differences did not translate into significant SBS differences. SEM likewise revealed only limited surface alteration after solvent application. This behavior may be interpreted in light of the previously reported composition of the tested DLP material, Asiga DentaTOOTH. According to the manufacturer, DentaTOOTH contains urethane dimethacrylate (UDMA), tetrahydrofurfuryl methacrylate (THFMA), and diphenyl(2,4,6-trimethylbenzoyl)phosphine oxide (TPO). In addition, a previous study on the uncured resin reported that UDMA-related species predominated, triethylene glycol dimethacrylate (TEGDMA) was also detected, and ethoxylated bisphenol A dimethacrylate (BisEMA)-related compounds were present at low abundance, with TPO identified as a common photoinitiator [32,33]. Such findings suggest that the tested DLP material behaved more like a highly cross-linked dimethacrylate-based resin than a PMMA-dominant substrate. Accordingly, the DLP material may have been less susceptible to solvent-induced swelling or superficial dissolution under the present conditions. In addition, the consistently high SBS observed in the DLP material may be partly related to favorable chemical compatibility between this methacrylate-based substrate and the adhesive/composite repair system used in this study. The universal adhesive contains MDP-based functional monomers, and the flowable composite is likewise based on methacrylate resin chemistry [23,34]. This may explain why the DLP material maintained high SBS regardless of solvent pretreatment.

The present findings were partly consistent with a recent in vitro study on aged CAD/CAM provisional restorative materials repaired with flowable resin composite, in which THF improved the repair bond strength of milled PMMA, whereas isopropyl alcohol showed limited benefit [15]. These observations were generally consistent with the present MIL results, in which THF improved repair performance relative to the control and IPA, and with the finding that some additively manufactured provisional materials may show greater repair potential than milled PMMA under appropriate conditions. However, the printed-material response differed between studies. In the previous study, the 3D-printed resin responded significantly to THF and MEK [15], whereas in the present study the tested DLP material showed the highest overall SBS but no significant differences among solvent pretreatments. This discrepancy may be related to differences in resin chemistry, aging protocol, and surface treatment design. The previous study evaluated thermocycled materials and included MEK and airborne-particle abrasion [15], whereas the present study used ACN and PYR and did not include long-term aging of the repaired interface. In addition, the present study evaluated AFM-based roughness from a limited scan area and predefined measurement sites, and the SEM observations were qualitative and based on representative specimens. Likewise, the previous study used a different roughness assessment method and also relied primarily on immediate bond-strength testing despite prior thermocycling of the substrates [15]. Therefore, direct numerical comparison between the two studies should be made with caution, and the findings are better interpreted in terms of general trends rather than exact equivalence. These considerations further support the interpretation that solvent effects on repair bonding are material-specific and should not be generalized across all CAD/CAM provisional restorative materials.

The SLA material requires a different interpretation because the tested substrate, Formlabs Premium Teeth, is a ceramic-filled resin rather than an unfilled polymeric resin [35]. In this material, solvent pretreatment would be expected to act primarily on the organic resin-rich superficial phase, whereas the ceramic filler fraction itself would remain largely unaffected. This helps explain why the roughness findings did not directly parallel the bond-strength results. In the present study, ACN produced the lowest SBS, whereas PYR produced the highest mean SBS. However, IPA showed higher S_a_ and S_q_ than THF and ACN, yet it did not provide the highest bond strength. The representative 3D AFM images were broadly consistent with the roughness findings, as the IPA-treated SLA surface showed more pronounced irregular topography than the THF- and ACN-treated surfaces, whereas THF and ACN showed comparatively more regular ridge-like features. SEM also suggested that THF and PYR produced relatively smoother and more homogeneous surfaces than ACN. These findings indicate that, in the ceramic-filled SLA resin, greater roughness was not necessarily associated with improved repair bonding. A plausible explanation is that an unfavorable solvent may selectively weaken the superficial resin matrix or create a weak boundary layer around filler-containing surface regions, thereby impairing stress transfer across the repaired interface. In contrast, a more suitable solvent may induce limited superficial modification while preserving the integrity of the filler–matrix surface structure.

The SEM observations and representative three-dimensional AFM images provided qualitative support for the AFM roughness findings, although the degree of agreement varied among materials. In the MIL material, the qualitative images broadly paralleled the quantitative AFM results, as solvent-treated surfaces showed more evident disruption of the original surface pattern together with higher S_a_ and S_q_ values than the control, and these changes were accompanied by improved SBS in the THF, ACN, and PYR groups. In contrast, this relationship was less clear in the SLA and DLP materials. In the SLA material, some solvent-treated surfaces appeared smoother or more homogeneous in SEM despite differences in roughness and bond strength, indicating that qualitative surface appearance did not always parallel repair performance. In the DLP material, only minor qualitative surface alterations were observed, which was broadly consistent with the absence of significant differences in S_a_ and the presence of only a limited pairwise difference in S_q_ among treatments, despite the consistently high SBS values. These findings suggest that qualitative surface imaging was useful for illustrating solvent-induced surface changes, but morphology alone could not predict repair bond strength across all materials.

Consistent with these observations, the relationship between roughness and repair bond strength was not universal across materials. In the MIL material, roughness and SBS broadly changed in the same direction for ACN and PYR, suggesting that solvent-mediated surface alteration was beneficial for repair, although THF improved SBS without a statistically significant increase in roughness. In contrast, this relationship was weaker in SLA and absent in DLP. The S_a_ and S_q_ values showed broadly similar trends across all groups, indicating consistency in the AFM-based roughness assessment. However, these roughness patterns did not always correspond to the bond-strength results, particularly in the SLA and DLP materials. These differences indicate that repair bonding in CAD/CAM provisional materials is controlled by multiple factors, including substrate chemistry, degree of cross-linking, solvent compatibility, preservation of superficial cohesive strength, and interaction with filler-containing surface regions [11,14]. Therefore, an increase in S_a_ or S_q_ should not be interpreted as a direct predictor of improved repair performance across all substrates.

The failure mode analysis generally supported the SBS findings, although the relationship was not identical across all groups. In the SLA material, the predominance of mixed failure suggested partial interfacial integrity despite the variability in bond strength. In the DLP material, the presence of mainly mixed failure together with some cohesive failure was consistent with its higher SBS values, indicating that fracture often extended beyond the adhesive interface. In the MIL material, however, failure mode did not fully parallel the SBS results. Although THF, ACN, and PYR improved bond strength relative to the control, the control, IPA, and THF groups still showed a relatively higher proportion of adhesive failure, whereas ACN and PYR showed more mixed failures. This suggests that THF was able to increase bond strength, but the interfacial failure pattern remained less favorable than that observed with ACN and PYR. Therefore, in the milled PMMA substrate, ACN and PYR appeared to provide not only improved repair bond strength but also a more favorable distribution of failure modes, indicating better interfacial integrity after repair.

From a clinical perspective, the present findings indicate that repair protocols for provisional restorations should be substrate-specific rather than universal. For milled PMMA, pretreatment with THF, ACN, or PYR appears beneficial. For the tested DLP material, additional solvent pretreatment may be unnecessary when a compatible adhesive–composite repair system is used. For the ceramic-filled SLA resin, solvent selection appears to be more critical, and ACN should be used with caution because it may adversely affect repair bond strength.

This study has several limitations. Only short-term bond strength was evaluated, and no thermocycling, fatigue loading, or long-term aging was performed. In addition, the exact proprietary formulations of the printed materials were not fully disclosed, which limits a more precise solubility-parameter analysis. Moreover, only one universal adhesive and one flowable resin composite were evaluated; therefore, the present findings should be interpreted within the context of the specific repair system used. SEM analysis was qualitative and based on one representative specimen per subgroup, and therefore the SEM findings should be interpreted as illustrative rather than fully representative of all specimens within each group. Similarly, although AFM provided quantitative roughness data, the limited scan area and predefined measurement sites may not have captured the full heterogeneity of the treated surfaces. Therefore, the solvent-compatibility explanation proposed here should be interpreted as a plausible mechanistic model rather than direct molecular proof. Further studies incorporating long-term aging and residual solvent analysis are needed to clarify how individual solvents influence the repairability of different CAD/CAM provisional materials. Furthermore, surface roughness was assessed solely by AFM, which provides high-resolution topographic data but is restricted to a relatively small scan area. Complementary techniques such as surface profilometry, which can sample larger areas, would provide a broader assessment of surface heterogeneity and should be considered in future studies. In addition, the present study evaluated only repair bond strength, whereas other clinically relevant mechanical properties of the repaired specimens, such as flexural strength, were not assessed. Future studies incorporating flexural strength testing and other mechanical evaluations of the repaired interface would help establish a more comprehensive understanding of repair performance in CAD/CAM provisional restorative materials.

In addition, although log transformation improved the suitability of the roughness data for parametric analysis, some deviation from residual normality remained, particularly for Sa. However, ANOVA F-tests have been reported to be robust to non-normality in terms of Type I error under variance homogeneity and a range of sample-size conditions [36]. Given the balanced design used in the present study (*n* = 5 per subgroup for roughness analysis), the parametric approach was therefore retained for inferential analysis. Nevertheless, the roughness findings should be interpreted with appropriate caution.

## 5. Conclusions

Within the limitations of this in vitro study, organic solvent pretreatment produced material-dependent effects on both surface roughness and repair bond strength of CAD/CAM provisional restorative materials. The tested DLP material showed the highest and most stable SBS overall, with no significant improvement after solvent pretreatment. In the MIL material, THF, ACN, and PYR significantly improved SBS, whereas roughness changes were most evident after ACN and PYR treatment; however, increased roughness did not consistently translate into higher bond strength. The ceramic-filled SLA material showed a more selective response, with ACN producing the lowest SBS and PYR producing the highest mean SBS. Overall, the relationship between surface roughness and repair bond strength was substrate-dependent, indicating that solvent selection and repair protocols should be tailored to the specific CAD/CAM provisional material.

## Figures and Tables

**Table 1:** Materials used in this study.

Category/Use	Product (Trade Name)	Manufacturer (Location)	Composition/Key Specification	LOT/Batch
CAD/CAM provisional material (milled PMMA)	Aidite Temp PMMA Blocks	Aidite (Qinhuangdao, Hebei, China)	Polymethyl methacrylate (PMMA), pigments	60523900
CAD/CAM provisional material (SLA-printed resin)	Formlabs Premium Teeth	Formlabs (Somerville, MA, USA)	Esterification products of 4,4′-isopropylidenediphenol, ethoxylated and 2-methylprop-2-enoic acid; diphenyl(2,4,6-trimethylbenzoyl)phosphine oxide	A301241004-02
CAD/CAM provisional material (DLP-printed resin)	ASIGA^®^ DentaTOOTH	ASIGA (Sydney, Australia)	7,7,9(or 7,9,9)-trimethyl-4,13-dioxo-3,14-dioxa-5,12-diazahexadecane-1,16-diyl bismethacrylate; tetrahydrofurfuryl methacrylate; diphenyl(2,4,6-trimethylbenzoyl)phosphine oxide	5000456
Universal adhesive	Scotchbond™ Universal Plus	3M ESPE (St. Paul, MN, USA)	10-MDP, HEMA, silane-treated silica, ethanol, water, camphorquinone, copolymer of acrylic and itaconic acid	12141223
Flowable resin composite	Filtek™ Supreme Flowable Restorative	3M ESPE (St. Paul, MN, USA)	Bis-GMA, UDMA, TEGDMA, Bis-EMA; filler particles	11845375

**Table 2:** Summary of two-way ANOVA for log-transformed shear bond strength (SBS), log-transformed arithmetic mean height (S_a_), and log-transformed root mean square height (S_q_).

Outcome	Source of Variation	df (Effect, Error)	F	*p*-Value
SBS (log-transformed)	Material	2, 135	154.1	<0.0001
	Solvent treatment	4, 135	14.20	<0.0001
	Material × solvent treatment	8, 135	20.04	<0.0001
S_a_ (log-transformed)	Material	2, 60	39.66	<0.0001
	Solvent treatment	4, 60	3.264	0.0173
	Material × solvent treatment	8, 60	4.407	0.0003
S_q_ (log-transformed)	Material	2, 60	41.53	<0.0001
	Solvent treatment	4, 60	3.540	0.0117
	Material × solvent treatment	8, 60	4.376	0.0003

*p* < 0.05 was considered statistically significant.

**Table 3:** Surface roughness (S_a_ and S_q_, mean ± SD, nm) of the experimental groups.

Materials	SLA		DLP		MIL	
	S_a_ (nm)	S_q_ (nm)	S_a_ (nm)	S_q_ (nm)	S_a_ (nm)	S_q_ (nm)
Control	187.31 ± 22.05 ^ab^	238.76 ± 25.03 ^ab^	141.97 ± 22.27 ^a^	186.56 ± 21.92 ^ab^	176.80 ± 21.15 ^a^	240.53 ± 34.59 ^a^
IPA	271.58 ± 13.10 ^a^	340.07 ± 21.84 ^a^	130.51 ± 49.20 ^a^	170.35 ± 65.71 ^ab^	297.97 ± 21.45 ^b^	381.94 ± 28.52 ^ab^
THF	154.05 ± 67.15 ^b^	205.47 ± 83.16 ^b^	166.61 ± 39.17 ^a^	222.83 ± 43.68 ^ab^	290.44 ± 40.72 ^ab^	372.82 ± 61.69 ^ab^
ACN	146.30 ± 42.87 ^b^	186.99 ± 57.56 ^b^	110.61 ± 31.62 ^a^	142.82 ± 38.61 ^a^	364.65 ± 67.28 ^b^	456.52 ± 70.72 ^b^
PYR	212.52 ± 74.71 ^ab^	275.72 ± 91.69 ^ab^	183.21 ± 61.85 ^a^	245.18 ± 77.57 ^b^	352.69 ± 110.58 ^b^	442.20 ± 127.58 ^b^

Values are presented as mean ± SD (*n* = 5 per subgroup). Within each material group and for each roughness parameter, values sharing the same lowercase superscript letter within the same column are not significantly different according to Tukey’s multiple comparisons test performed on the log-transformed data (*p* > 0.05).

## Data Availability

The data presented in this study are contained within the article and Appendix A. Further inquiries can be directed to the corresponding author.

## Abbreviations

The following abbreviations are used in this manuscript:

SBS	Shear bond strength
CAD/CAM	Computer-Aided Design/Computer-Aided Manufacturing
PMMA	Polymethyl methacrylate
SLA	Stereolithography
DLP	Digital light processing
SEM	Scanning electron microscopy
IPA	Isopropyl alcohol
THF	Tetrahydrofuran
ACN	Acetonitrile
PYR	Pyridine
UDMA	Urethane dimethacrylate
THFMA	Tetrahydrofurfuryl methacrylate
TPO	Diphenyl(2,4,6-trimethylbenzoyl)phosphine oxide
TEGDMA	Triethylene glycol dimethacrylate
BisEMA	Ethoxylated bisphenol A dimethacrylate

## Appendix A

### Appendix A.1

**Table A1:** Brown–Forsythe test results for homogeneity of variance of untransformed and log-transformed SBS, S_a_, and S_q_ data.

Outcome	Brown–Forsythe Statistic	df1	df2	*p*-Value
SBS (untransformed)	2.762	14	135	0.001
SBS (log-transformed)	0.505	14	135	0.927
S_a_ (untransformed)	2.076	14	60	0.026
S_a_ (log-transformed)	1.199	14	60	0.300
S_q_ (untransformed)	1.883	14	60	0.047
S_q_ (log-transformed)	1.284	14	60	0.244

Brown–Forsythe test results are reported using the median-based formulation. *p* < 0.05 indicates significant heterogeneity of variance.

### Appendix A.2

**Table A2:** Shear bond strength values (mean ± SD, MPa) of the tested materials after different solvent pretreatments.

	Shear Bond Strength (MPa)		
	SLA	DLP	MIL
Control	10.12 ± 1.99 ^ab^	16.65 ± 3.85 ^a^	5.14 ± 1.32 ^a^
IPA	8.24 ± 1.33 ^bc^	13.77 ± 4.06 ^a^	3.67 ± 0.76 ^b^
THF	7.43 ± 2.04 ^c^	15.18 ± 3.06 ^a^	9.51 ± 2.61 ^c^
ACN	5.13 ± 1.31 ^d^	17.86 ± 4.26 ^a^	10.12 ± 1.80 ^c^
PYR	11.48 ± 2.69 ^a^	15.22 ± 2.90 ^a^	9.57 ± 2.70 ^c^

Values are presented as mean ± SD (*n* = 10 per subgroup). Different lowercase letters within the same column indicate significant differences among solvent treatments within the same material group (Tukey’s post hoc test, *p* < 0.05).

### Appendix A.3

**Table A3:** Hansen solubility parameters of solvents and materials used in this study.

Solvent	δD (Dispersion)	δP (Polar)	δH (H-Bonding)	δT (Total)
Isopropyl alcohol	15.8	6.1	16.4	22.7
Tetrahydrofuran	16.8	5.7	8.0	21.8
Acetonitrile	15.3	18.0	6.1	23.5
Pyridine	19.0	8.8	5.9	24.3
PMMA	18.6	10.5	7.5	22.7
